# Successful management of anaphylactic shock prior to elective cesarean delivery: a case report

**DOI:** 10.1186/s40981-024-00750-z

**Published:** 2024-10-26

**Authors:** Kaede Watanabe, Nazuha Mohd Najid, Yusuke Mazda

**Affiliations:** 1 Department of Obstetric Anesthesiology, Center for Maternal-Fetal and Neonatal Medicine, Saitama Medical Center, Saitama Medical University, Kawagoe, Japan; 2 Department of Anesthesiology and Critical Care, Hospital Sultanah Bahiyah Alor Setar, Kedah, Malaysia

**Keywords:** Anaphylactic shock, Cesarean delivery, Perioperative antibiotics

## Abstract

**Background:**

Anaphylactic cardiovascular collapse is complicated by aortocaval compression during pregnancy, exacerbated by neuraxial anesthesia. Despite recommendations to administer perioperative antibiotics before anesthesia, common practice in Japan involves administering them after anesthesia induction. We report a case of possible antibiotics-induced anaphylaxis just before anesthesia for cesarean delivery.

**Case presentation:**

A 24-year-old woman at 37 weeks of gestation presented for a scheduled repeat cesarean under spinal anesthesia. After starting administration of cefazolin prior to anesthesia, she developed anaphylactic symptoms. Hypotension refractory to adrenaline necessitated conversion to an emergency cesarean section under general anesthesia. A neonate was delivered with favorable Apgar scores. Post-delivery, the mother’s hemodynamics stabilized significantly. Elevated plasma tryptase confirmed anaphylaxis. Both mother and infant were discharged without further complications.

**Conclusions:**

This case emphasizes the importance of administering prophylactic antibiotic before anesthesia in mitigating severity of shock induced by anaphylaxis and the crucial role of prompt emergency cesarean in achieving successful outcomes.

## Background

Anaphylaxis in pregnancy is a rare but potentially severe complication affecting both the mother and the baby. According to recent population-based data, the incidence of anaphylaxis in pregnancy is 1.5 per 100,000 maternities, with one in seven cases resulting in poor outcomes for both the mothers and babies [1]. Managing anaphylaxis during the third trimester of pregnancy can be particularly challenging due to the combined effects of aortocaval compression and cardiovascular disturbances associated with anaphylaxis. Additionally, anaphylaxis-induced hypotension is likely to be exacerbated using neuraxial anesthesia. Since antibiotics are the most common cause of anaphylaxis perioperatively [2], there is an argument for antibiotics to be administered several minutes before the induction of anesthesia [3]. In this report, we present a case of possible antibiotic-induced anaphylaxis that occurred prior to spinal anesthesia for an elective cesarean delivery. Hypotension refractory to adrenaline prompted a resuscitative cesarean delivery under general anesthesia, leading to a successful outcome.

## Case presentation

A 24-year-old, gravida 5, para 1 woman was scheduled for repeat cesarean section at 37 weeks of gestation under spinal anesthesia. Her body weight and height before cesarean delivery were 50.3 kg and 155 cm, respectively. She had a history of prepartum severe ileus that necessitated an urgent cesarean delivery 4 years prior. Her medical history included well-controlled panic disorder, with clomipramine and bromazepam. Additionally, she had undergone an uneventful right meniscus repair surgery under general anesthesia 1 year ago. Although she had a history of hay fever, she had no recorded food or drug allergies.

**Fig. 1:**
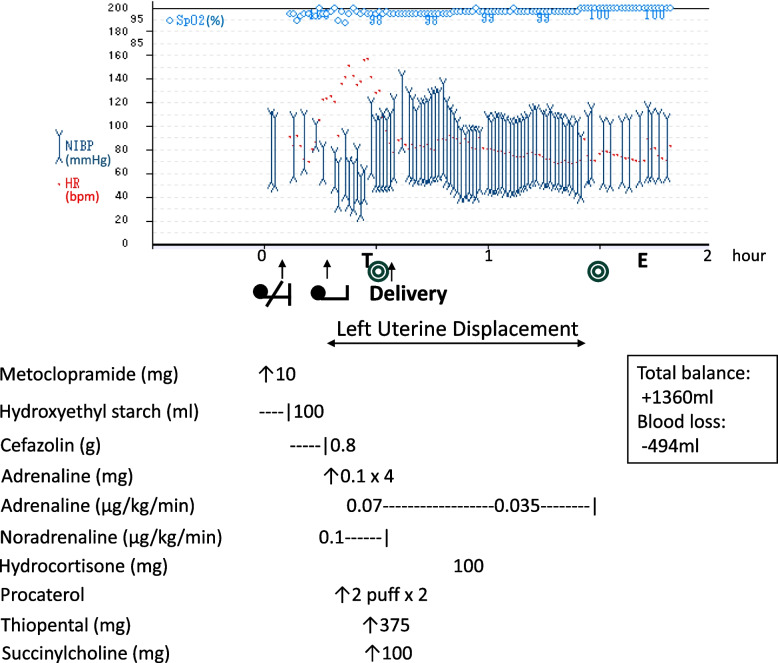
Anesthesia record. The patient complained of itchiness and developed a systemic eruption 3 min after starting cefazolin infusion, followed by dyspnea, hypotension, and tachycardia. She was repositioned to a supine position with left uterine displacement. Intravenous adrenaline was administered repeatedly, along with procaterol aerosol for dyspnea and a continuous infusion of noradrenaline. Despite these measures, her vital signs remained unstable, and fetal bradycardia prompted an emergency cesarean section under general anesthesia. A healthy newborn was delivered 20 min after the onset of anaphylaxis. The mother’s condition stabilized significantly post-delivery, allowing for weaning off catecholamine infusions. Hydrocortisone was administered to prevent further allergic reactions. The total blood loss was 494 mL with a fluid balance of + 1360 mL. Abbreviations: SpO_2_, arterial oxygen saturation; NIBP, non-invasive blood pressure; HR, heart rate; Time 0, patient’s entrance into the operating theater; T, tracheal intubation; E, extubation

Upon her arrival in the operating room, routine monitoring was initiated, and intravenous administration of 6% hydroxyethyl starch solution was started. Following intravenous administration of metoclopramide, the patient was positioned in the right lateral decubitus posture in preparation for spinal anesthesia, and infusion of cefazolin 1 g was started. After infusion of 0.8 g cefazolin in 3 min and before sterilization of the patients’ back, she reported skin itchiness. Physical examination revealed erythema across her entire body. Blood pressure dropped to 83/57 mmHg; heart rate increased to 124 bpm. Peripheral oxygen saturation remained at 98% in room air (Fig. 1).

Promptly, supplemental oxygen was initiated, and 0.1 mg of intravenous adrenaline was administered 10 min after the onset of symptoms. The patient was repositioned to a supine position with left uterine displacement, and intravenous adrenaline 0.1 mg was repeated. The patient then complained of dyspnea, and procaterol aerosol was given for relief. Additionally, a continuous infusion of noradrenaline at 0.1 μg/kg/min was initiated. Despite these interventions, her vital signs remained unstable, with blood pressure ranging 60–80/30–40 mmHg and heart rate 140–150 bpm. Fetal bradycardia was detected through Doppler fetal heart rate monitoring, necessitating the decision for an emergency cesarean section under general anesthesia.

After rapid sequence induction using thiopental 375 mg, succinylcholine 100 mg, and tracheal intubation, cesarean section was started. A female baby was delivered 20 min after the onset of anaphylaxis symptoms, with Apgar scores of 7 at 1 min and 9 at 5 min. The umbilical artery pH was measured at 7.141, PCO_2_ 73.2 mmHg, base excess − 4.0 mmol/L, HCO_3_^−^ 25.2 mmol/L, and lactate 5.9 mmol/L. A brief period of continuous positive airway pressure was applied to assist with breathing via a face mask, which resulted in a quick recovery.

Immediately after delivery, the vital signs began to recover and stabilize. The requirement for catecholamine support gradually lessened, becoming unnecessary by the end of the surgery. Hydrocortisone 100 mg was administered to prevent the recurrence of an acute allergic reaction. Sevoflurane, midazolam, and morphine were used for maintenance of anesthesia. Total blood loss during the surgery was 494 mL, and total fluid balance was + 1360 mL.

She remained under observation in the intensive care unit overnight, and no subsequent event occurred. The baby was observed overnight in the neonatal intensive care unit, experiencing no adverse events. On the fifth day post-surgery, the mother and her baby were discharged home.

Plasma histamine levels were within the normal range 30 min after symptom onset, likely due to its rapid degradation, as indicated by the decreasing trend over time. Notably, however, the plasma tryptase level showed a significant increase at 30 min and 2 h after symptom onset (Table 1). The significant rise in tryptase levels, along with the clinical course, confirmed a definitive diagnosis of anaphylaxis.

**Table 1:** Plasma histamine and tryptase levels

**Time from onset**	**Histamine (ng/mL)**
30 min	0.79
2 hr	0.47
24 hr	0.42
Reference value: 0.15-1.23	
**Time from onset**	**Tryptase (µg/L)**
30 min	15.7
2 hr	18.3
24 hr	3.6

The mother’s plasma histamine levels were within the normal range 30 minutes after symptom onset, likely due to its rapid degradation, as indicated by the decreasing trend over time. Notably, the plasma tryptase levels were significantly elevated at 30 minutes (15.7 µg/L) and 2 hours (18.3 µg/L) after symptom onset, confirming the diagnosis of anaphylaxis. There are several criteria on the interpretation of plasma tryptase levels. For example, an elevated plasma tryptase level, a percentage change >141% compared with baseline, >1.2x baseline +2, or an absolute tryptase level of >15.7 µg/L has been reported to be highly predictive of perioperative anaphylaxis [4, 5]

We had intended to conduct an allergy examination to ascertain the possible trigger of the anaphylaxis (cefazolin, hydroxyethyl starch, or metoclopramide). However, scheduling difficulties have prevented her from attending our clinic up to this point.

## Discussion

We experienced maternal anaphylaxis shock that was likely induced by cefazolin before spinal anesthesia. The patient was successfully resuscitated through prompt cesarean delivery. Despite the absence of allergen testing for possible trigger of anaphylaxis, we strongly suspect cefazolin as the causative agent, given its known propensity to induce allergies and the alignment of the clinical presentation in this case. Maternal anaphylaxis shock induced by prophylactic antibiotics associated with cesarean delivery is a rare event, with the incidence of 2.1 (95% CI: 1.1–3.6) per 100,000 cesarean deliveries [6]. However, it is important for anesthesiologists to be aware of the possibility. Anaphylaxis induced by hydroxyethyl starch (HES) or metoclopramide is exceedingly rare compared to that caused by antibiotics, with most data derived from case reports. Although one observational study identified HES as a common suspected trigger of perioperative allergic reactions, the lack of confirmatory allergy tests leaves the true incidence of HES-induced anaphylaxis uncertain [7]. Given its frequent use as an infusion solution during anesthesia, HES may often be listed as a suspected trigger; however, its actual incidence as a cause of anaphylaxis is likely very low.

In our case, the administration of cefazolin prior to spinal anesthesia appears to have played a crucial role in achieving favorable outcomes, because anaphylaxis-induced hypotension could have been exacerbated by the concurrent neuraxial blockade. The National Institute of Academic Anaesthesia recommends the early administration of perioperative antibiotics several minutes before induction of anesthesia [3]. Given their findings that signs of anaphylaxis are identified in less than 5 min in most cases, and in almost all cases within 10 min after receiving antibiotics, it would be ideal to administer perioperative antibiotics at least 5–10 min before the induction of anesthesia. Importantly, this proactive approach does not compromise the efficacy of antibiotics. However, in practice, antibiotics are often administered after the induction of anesthesia in Japan. A previous Japanese multicenter retrospective observational study on anaphylaxis during general anesthesia settings showed that anaphylaxis events induced by antibiotics were all detected during the maintenance of anesthesia, and the average time to the first symptom was as late as 5–10 min after administering the causative drugs [8]. As observed in our case, we were able to detect the anaphylaxis symptoms immediately after their onset, avoiding confusion with spinal block-induced hypotension. Administering antibiotics before anesthesia induction can facilitate the rapid identification and management of anaphylactic reactions, potentially reducing the risk of cardiopulmonary collapse.

Following the cesarean delivery, we observed a rapid recovery of the patient’s vital signs. In our case, where maternal cardiovascular instability persisted despite adequate anaphylaxis treatment, the prompt induction of general anesthesia for immediate delivery was likely beneficial, even though inducing general anesthesia during a cardiopulmonary crisis presents significant challenges, such as airway management difficulties and potential cardiovascular decompensation. This experience aligns with previous recommendations that cesarean delivery is beneficial in case of severe anaphylaxis with persistent maternal hemodynamic instability despite resuscitation efforts [9]. Cesarean delivery in this scenario could be beneficial for both the mother and baby. As previously reported, emptying the uterus removes aortocaval compression, resulting in 60% to 80% increase in cardiac output [9]. Current maternal cardiopulmonary resuscitation guidelines recommend resuscitative hysterotomy and immediate delivery of fetus in case of maternal cardiac arrest when initial resuscitation does not rapidly result in return of spontaneous circulation [10, 11]. Additionally, during maternal anaphylaxis, appropriate placental perfusion and fetal oxygenation is not guaranteed. Prompt cesarean delivery potentially reduce the risk of hypoxic injury in the infant, particularly when persistent non-reassuring fetal heart rate patterns are observed [9]. It is crucial to remember that cesarean delivery can be a valid therapeutic option in the management of maternal anaphylactic shock.

A limitation of this case report is the absence of allergen testing. Although the incidence of anaphylaxis induced by hydroxyethyl starch or metoclopramide is extremely rare compared to antibiotics, the actual trigger of the anaphylaxis remains unidentified. It is important for future research to include comprehensive allergy testing to accurately determine the trigger of anaphylactic symptoms.

## Conclusions

This case of maternal anaphylaxis highlights the potential benefits of administering prophylactic antibiotic before anesthesia, as it may help mitigate the severity of physiological derangement associated with anaphylaxis. Further research is necessary to validate these findings, particularly in complicated cases. The prompt and efficient response, including the timely decision to perform an emergency cesarean section, proved instrumental in saving the lives of both the mother and the baby.

## Data Availability

The datasets used during the current study are available from the corresponding author on reasonable request.
